# Supplementary material for: mRNA cap-binding protein eIF4E1 is a novel regulator of Toxoplasma gondii latency
Source: mBio. 2024 May 15;15(6):e02954-23. doi: 10.1128/mbio.02954-23 (PMC11237481; doi:10.1128/mbio.02954-23)
Supplement: Supplemental Figures — Figures S1-S6. [file mbio.02954-23-s0001.pdf]

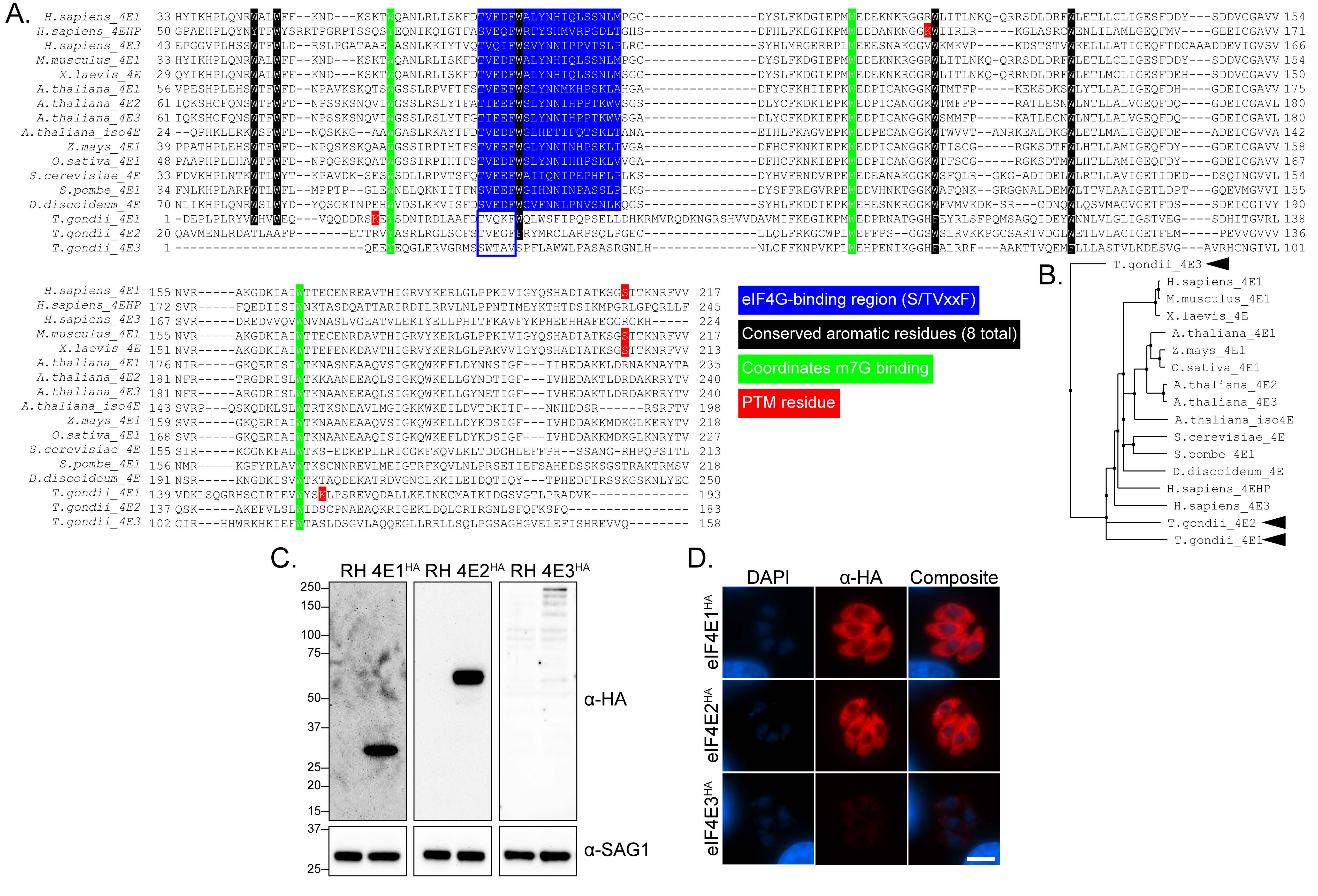
A.
H.sapiens_4E1 33 HYIKHPLQNRWALWFF--KND---KSKTWQANLRLISKFDTVEDFWALYNHIQLSSNLMPGC---------------DYSLFKDGIEPMWEDEKNKRGGRWLITLNKQ-QRRSDLDRFWLETLLCLIGESFDDY---SDDVCGAVV 154
H.sapiens_4EHP 50 GPAEHPLQYNYTFWYSRRTPGRPTSSQSYEQNIKQIGTFASVEQFWRFYSHMVRPGDLTGHS---------------DFHLFKEGIKPMWEDDANKNGGKWIIRLR-----KGLASRCWENLILAMLGEQFMV----GEEICGAVV 171
H.sapiens_4E3 43 EPGGVPLHSSWTFWLD--RSLPGATAAECASNLKKIYTVQTVQIFWSVYNNIPPVTSLPLRC---------------SYHLMRGERRPLWEEESNAKGGVWKMKVP-----KDSTSTVWKELLLATIGEQFTDCAAADDEVIGVSV 166
M.musculus_4E1 33 HYIKHPLQNRWALWFF--KND---KSKTWQANLRLISKFDTVEDFWALYNHIQLSSNLMPGC---------------DYSLFKDGIEPMWEDEKNKRGGRWLITLNKQ-QRRSDLDRFWLETLLCLIGESFDDY---SDDVCGAVV 154
X.laevis_4E 29 QYIKHPLQNRWALWFF--KND---KSKTWQANLRLISKFDTVEDFWALYNHIQLSSNLMSGC---------------DYSLFKDGIEPMWEDEKNKRGGRWLITLNKQ-QRRNDLDRFWLETLMCLIGESFDEH---SDDVCGAVV 150
A.thaliana_4E1 56 VPESHPLEHSWTFWFD-NPAVKSKQTSWGSSLRPVFTFSTVEEFWSLYNNMKHPSKLAHGA---------------DFYCFKHIIEPKWEDPICANGGKWTMTFP-----KEKSDKSWLYTLLALIGEQFDH----GDEICGAVV 175
A.thaliana_4E2 61 IQKSHCFQNSWTFWFD--NPSSKSNQVIWGSSLRSLYTFATIEEFWSLYNNIHPPTKWVSGS---------------DLYCFKDKIEPKWEDPICANGGKWTMFFP-----RATLESNWLNTLLALVGEQFDQ----GDEICGAVL 180
A.thaliana_4E3 61 IQKSHCFQNSWTFWFD--NPSSKSNQVIWGSSLRSLYTFGTIEEFWSLYNNIHPPTKWVSGA---------------DLYCFKDKIEPKWEDPICANGGKWSMMFP-----KATLECNWLNTLLALVGEQFDQ----GDEICGAVL 180
A.thaliana_iso4E 24 --QPHKLERKWSFWFD--NQSKKG--AAWGASLRKAYTFDTVEDFWGLHETIFQTSKLTANA---------------EIHLFKAGVEPKWEDPECANGGKWTWVVT--ANRKEALDKGWLETLMALIGEQFDE----ADEICGVVA 142
Z.mays_4E1 39 PPATHPLEHSWTFWFD--NPQSKSKQAAWGSSIRPIHTFSTVEEFWGLYNNINHPSKLIVGA---------------DFHCFKNKIEPKWEDPICANGGKWTISCG-----RGKSDTFWLHTLLAMIGEQFDY----GDEICGAVV 158
O.sativa_4E1 48 PAAPHPLEHAWTFWFD--NPQGKSKQATWGSSIRPIHTFSTVEDFWSLYNNIHHPSKLVVGA---------------DFHCFKNKIEPKWEDPICANGGKWTFSCG-----RGKSDTMWLHTLLAMIGEQFDY----GDEICGAVV 167
S.cerevisiae_4E 33 FDVKHPLNTKWTLWYT--KPAVDK-SESWSDLLRPVTSFQTVEEFWAIIQNIPEPHELPLKS---------------DYHVFRNDVRPEWEDEANAKGGKWSFQLR--GKG-ADIDELWLRTLLAVIGETIDED---DSQINGVVL 154
S.pombe_4E1 34 FNLKHPLARPWTLWFL--MPPTP--GLEWNELQKNIITFNSVEEFWGIHNNINPASSLPIKS---------------DYSFFREGVRPEWEDVHNKTGGKWAFQNK--GRGGNALDEMWLTTVLAAIGETLDPT---GQEVMGVVI 155
D.discoideum_4E 70 NLIKHPLQNRWSLWYD--YQSGKINPEHWVDSLKKVISFDSVEDFWCVFNNLPNVSNLKQGS---------------SYHLFKDDIEPKWEHESNKRGGKWFVMVKDK-SR---CDNQWLQSVMACVGETFDS----SDEICGIVY 190
T.gondii_4E1 1 -DEPLPLRYVWHVWEQ---VQQDDRSKEYSDNTRDLAAFDTVQKFWQLWSFIPQPSELLDHKRMVRQDKNGRSHVVDAVMIFKEGIKPMWEDPRNATGGHFEYRLSFPQMSAGQIDEYWNNLVLGLIGSTVEG----SDHITGVRL 138
T.gondii_4E2 20 QAVMENLRDATLAAFP-------ETTRVYASRLRGLSCFSTVEGFFRYMRCLARPSQLPGEC---------------LLQLFRKGCWPLWEFFPS--GGSWSLRVKKPGCSARTVDGLWETLVLACIGETFEM-----PEVVGVVV 136
T.gondii_4E3 1 -------------------------QEEYEQGLERVGRMSSWTAVSPFLAWWLPASASRGNLH---------------NLCFFKNPVKPLWEHPENIKGGHFALRRF---AAKTTVQEMFLLLASTVLKDESVG---AVRHCNGIVL 101
H.sapiens_4E1 155 NVR-----AKGDKIAIWTTECENREAVTHIGRVYKERLGLPPKIVIGYQSHADTATKSGSTTKNRFVV 217
H.sapiens_4EHP 172 SVR-----FQEDIISIWNKTASDQATTARIRDTLRRVLNLPPNTIMEYKTHTDSIKMPGRLGPQRLLF 245
H.sapiens_4E3 167 SVR-----DREDVVQVWNVNASLVGEATVLEKIYELLPHITFKAVFYKPHEEHHAFEGGRGKH----- 224
M.musculus_4E1 155 NVR-----AKGDKIAIWTTECENRDAVTHIGRVYKERLGLPPKIVIGYQSHADTATKSGSTTKNRFVV 217
X.laevis_4E 151 NVR-----AKGDKIAIWTTEFENKDAVTHIGRVYKERLGLPAKVVIGYQSHADTATKSGSTTKNRFVV 213
A.thaliana_4E1 176 NIR-----GKQERISIWTKNASNEAAQVSIGKQWKEFLDYNNSIGF---IIHEDAKKLDRNAKNAYTA 235
A.thaliana_4E2 181 NFR-----TRGDRISLWTKKAANEEAQLSIGKQWKELLGYNDTIGF---IVHEDAKTLDRDAKRRYTV 240
A.thaliana_4E3 181 NFR-----ARGDRISLWTKNAANEEAQLSIGKQWKELLGYNETIGF---IVHEDAKTLDRDAKRRYTV 240
A.thaliana_iso4E 143 SVRP---QSKQDKLSLWTRTKSNEAVLMGIGKKWKEILDVTDKITF---NNHDDSR------RSRFTV 198
Z.mays_4E1 159 SVR-----GKQERIAIWTKNAANEAAQVSIGKQWKELLDYKDSIGF---IVHDDAKKMDKGLKERYTV 218
O.sativa_4E1 168 SVR-----GKQERIAIWTKNAANEAAQISIGKQWKEFLDYKDSIGF---IVHDDAKKMDKGLKNRYTV 227
S.cerevisiae_4E 155 SIR-----KGGNKFALWTKS-EDKEPLLRIGGKFKQVLKLTDDGHLEFFPH--SSANG-RHPQPSITL 213
S.pombe_4E1 156 NMR-----KGFYRLAVWTKSCNNREVLMEIGTRFKQVLNLPRSETIEFSAHEDSSKSGSTRAKTRMSV 218
D.discoideum_4E 191 NSR-----KNGDKISVWTKTAQDEKATRDVGNCLKKILEIDQTIQY---TPHEDFIRSSKGSKNLYEC 250
T.gondii_4E1 139 VDKLSQGRHSCIRIEVWYSKLPSREVQDALLKEINKCMATKIDGSVGTLPRADVK------------- 193
T.gondii_4E2 137 QSK-----AKEFVLSLWIDSCPNAEAQKRIGEKLDQLCRIRGNLSFQFKSFQ---------------- 183
T.gondii_4E3 102 CIR---HHWRKHKIEFWTASLDSGVLAQQEGLLRRLLSQLPGSAGHGVELEFISHREVVQ-------- 158
eIF4G-binding region (S/TVxxF)
Conserved aromatic residues (8 total)
Coordinates m7G binding
PTM residue
B.
T.gondii_4E3
H.sapiens_4E1
M.musculus_4E1
X.laevis_4E
A.thaliana_4E1
Z.mays_4E1
O.sativa_4E1
A.thaliana_4E2
A.thaliana_4E3
A.thaliana_iso4E
S.cerevisiae_4E
S.pombe_4E1
D.discoideum_4E
H.sapiens_4EHP
H.sapiens_4E3
T.gondii_4E2
T.gondii_4E1
C.
RH 4E1HA RH 4E2HA RH 4E3HA
250
150
100
75
50
37
25
20
15
37
25
α-HA
α-SAG1
D.
DAPI
α-HA
Composite
eIF4E1HA
eIF4E2HA
eIF4E3HA

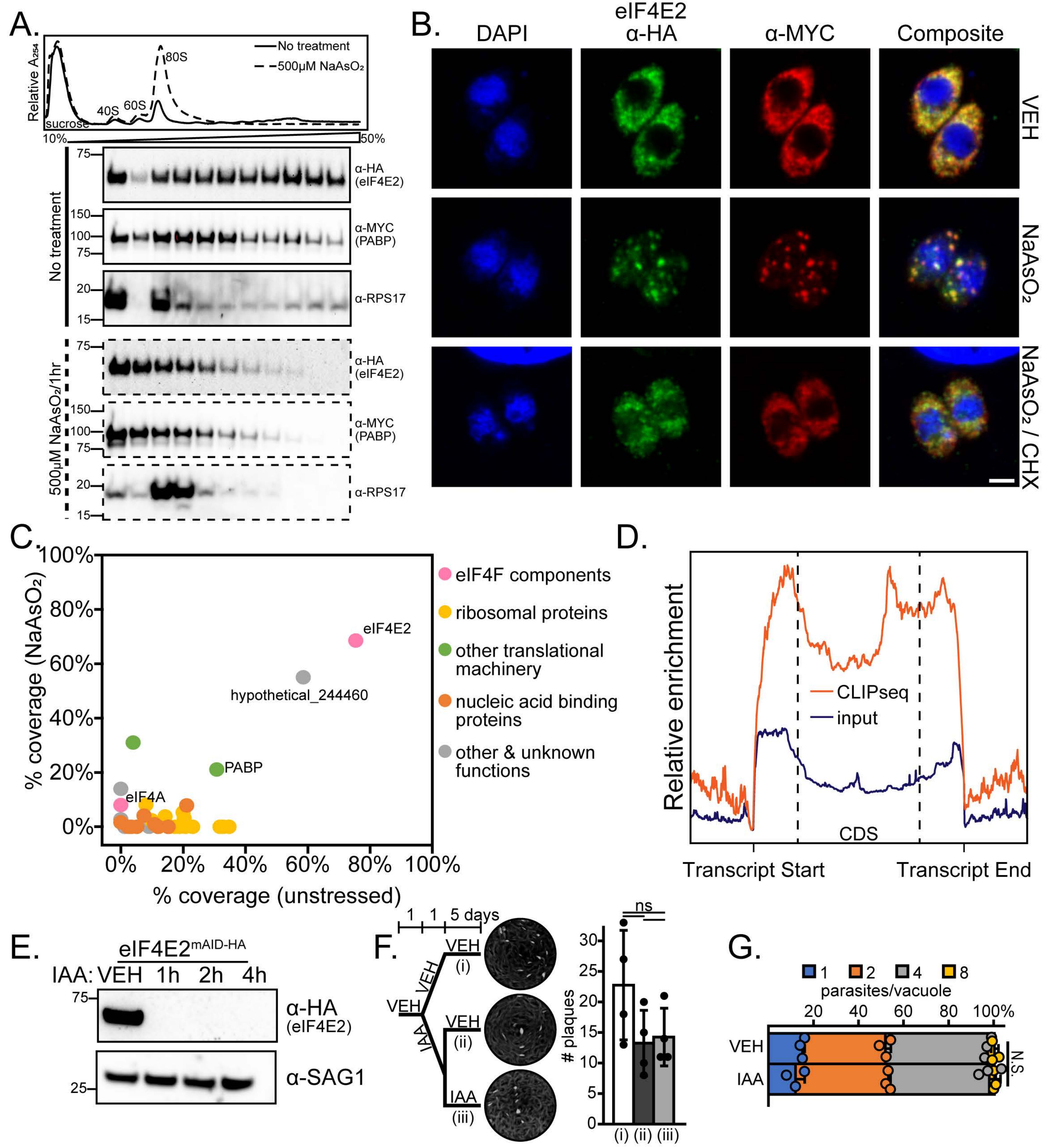
A.
Relative A254
No treatment
500μM NaAsO2
80S
40S
60S
sucrose
10%
50%
No treatment
500μM NaAsO2/1hr
75
150
100
20
15
α-HA (eIF4E2)
α-MYC (PABP)
α-RPS17
B.
DAPI
eIF4E2 α-HA
α-MYC
Composite
VEH
NaAsO2
NaAsO2 / CHX
C.
% coverage (NaAsO2)
% coverage (unstressed)
0%
20%
40%
60%
80%
100%
eIF4E2
hypothetical_244460
PABP
eIF4A
eIF4F components
ribosomal proteins
other translational machinery
nucleic acid binding proteins
other & unknown functions
D.
Relative enrichment
CLIPseq
input
CDS
Transcript Start
Transcript End
E.
eIF4E2mAID-HA
IAA: VEH 1h 2h 4h
75
25
α-HA (eIF4E2)
α-SAG1
F.
1 1 5 days
VEH
IAA
VEH (i)
VEH (ii)
IAA (iii)
plaques
ns
0
5
10
15
20
25
30
(i) (ii) (iii)
G.
1 2 4 8
parasites/vacuole
20
40
60
80
100%
VEH
IAA
N.S.

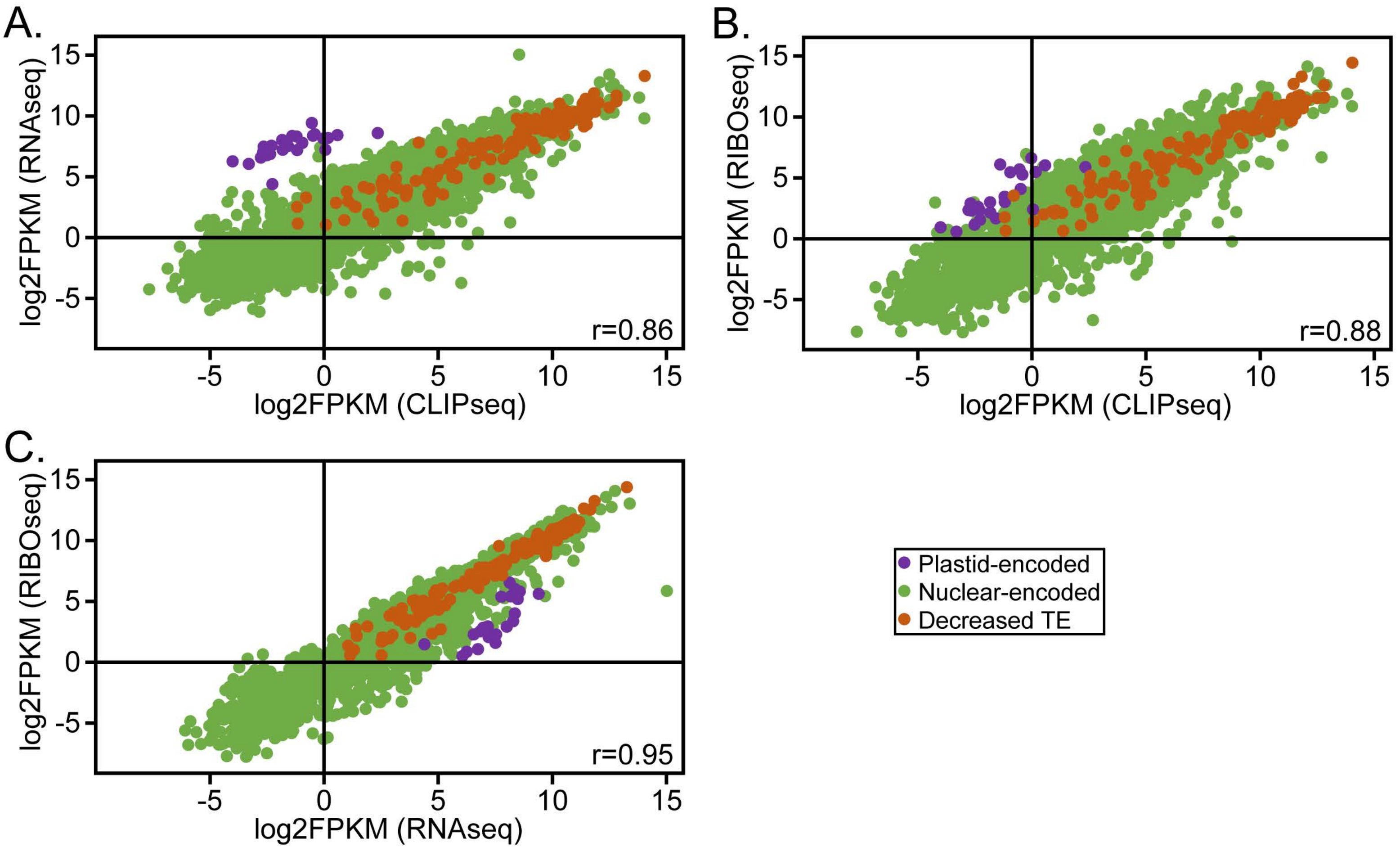

A.
B.
C.
log2FPKM (RNAseq)
log2FPKM (CLIPseq)
log2FPKM (RIBOseq)
r=0.86
r=0.88
r=0.95
15
10
5
0
-5
Plastid-encoded
Nuclear-encoded
Decreased TE

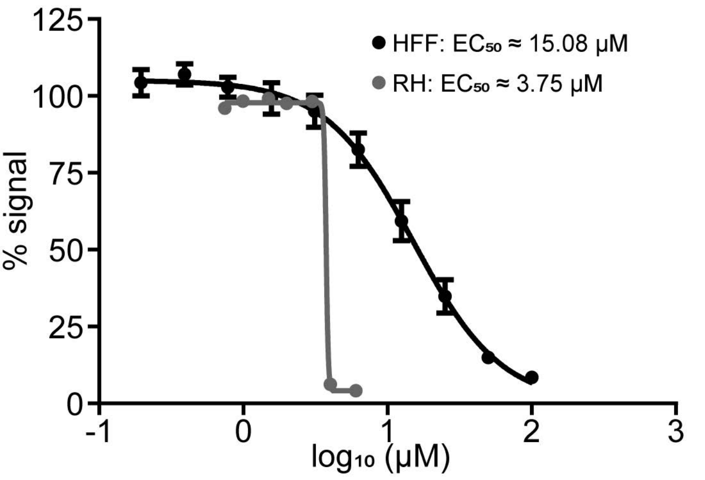

HFF: EC50 ≈ 15.08 µM
RH: EC50 ≈ 3.75 µM
125
100
75
50
25
0
% signal
-1
0
1
2
3
log10 (µM)

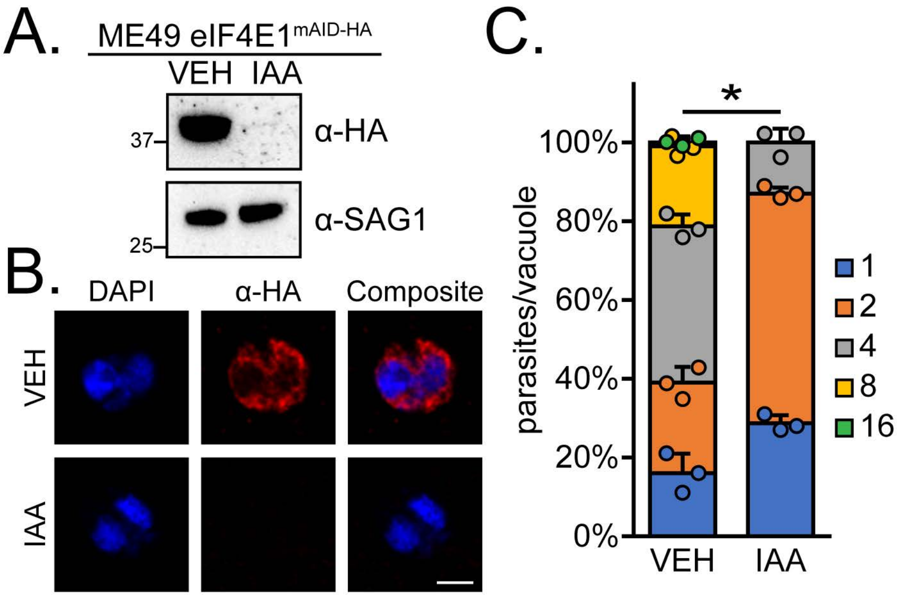
A.
ME49 eIF4E1mAID-HA
VEH
IAA
37
25
α-HA
α-SAG1
B.
DAPI
α-HA
Composite
VEH
IAA
C.
*
parasites/vacuole
100%
80%
60%
40%
20%
0%
VEH
IAA
1
2
4
8
16

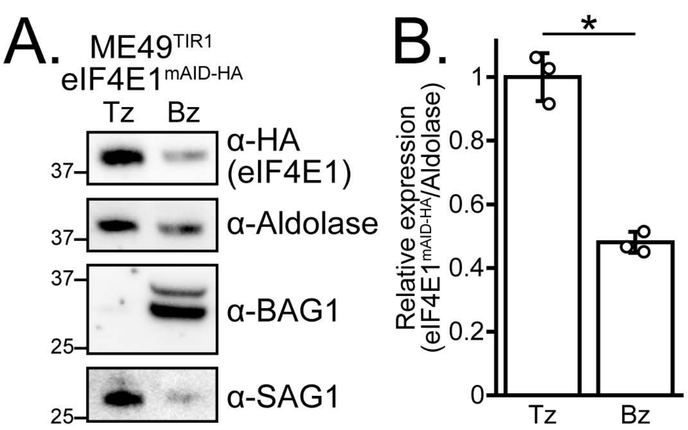
A.
ME49^{TIR1}
eIF4E1$^{mAID-HA}$
Tz
Bz
α-HA
(eIF4E1)
37
α-Aldolase
37
37
α-BAG1
25
α-SAG1
25
B.
*
Relative expression
(eIF4E1$^{mAID-HA}$/Aldolase)
1
0.8
0.6
0.4
0.2
0
Tz
Bz
