# Supplementary material for: mRNA cap-binding protein eIF4E1 is a novel regulator of Toxoplasma gondii latency
Source: mBio. 2024 May 15;15(6):e02954-23. doi: 10.1128/mbio.02954-23 (PMC11237481; doi:10.1128/mbio.02954-23)
Supplement: Legends — for supplemental figures and tables. [file mbio.02954-23-s0002.docx]

# Supplemental Figure Legends

**Figure S1. The *Toxoplasma* eIF4E family members retain highly conserved features of the eIF4E domain.** A) Multiple sequence alignment (MUSCLE) of eIF4E domains from multiple model species along with the three *Toxoplasma* orthologs. Motifs responsible for eIF4G-binding and key conserved aromatic residues are highlighted and defined in the color-coded index. B) Phylogram of the multiple sequence alignment. C) Western blot of endogenously tagged eIF4E paralogs. The parental strain, RHΔKu80ΔHX, is denoted as RH in the first lane of each blot. SAG1 was probed as a loading control. Molecular weight markers are indicated in kDa. D) Immunofluorescence microscopy of each HA-tagged eIF4E paralog. DNA is stained with DAPI. Microscopy scale bar = 5 µm.

**Figure S2. eIF4E2 is dispensable for tachyzoite fitness and does not operate within an eIF4F complex.** A) Analysis of eIF4E2^HA^ and PABP^MYC^ sedimentation by polysome profiling of tachyzoites exposed to oxidative stress induced by incubation with 500 µM NaAsO_2_ for 1 h or no treatment. The trace is generated by continuous absorbance at A254 which measures RNA content with major peaks at rRNA subunits, monosome, and heavy polysomes. Fractions of equal volumes were collected from the gradient and proteins were enriched by TCA precipitation and analyzed by western blot. An antibody directed against RPS17 that is suggested to recognize both *Toxoplasma* and human orthologs was included on the blot as a reference marker for protein sedimentation profiles. B) Analysis of eIF4E2 localization to stress granules upon oxidative stress. eIF4E2^HA^ and PABP1^MYC^ were visualized by immunofluorescence microscopy and DNA by DAPI staining. Samples were treated with 500 µM NaAsO_2_ ± 100 µg/ml cycloheximide (CHX) for 1 h prior to fixation. The translation elongation inhibitor CHX was included as a control to prevent stress granule formation. Microscopy scale bar = 2 µm. C) eIF4E2^HA^ interactomes under stressed (y-axis; 500 µM NaAsO_2_ for 1 h) or non-stressed (x-axis) tachyzoites. Affinity purification of eIF4E2^HA^ was conducted by immunoprecipitation with α-HA magnetic beads followed by mass spectrometry identification from two biological replicates. Interacting proteins that were absent in the untagged control immunoprecipitations and not localized to *Toxoplasma* secretory organelles (43) are displayed and colored by functional identity. The average percentage of coverage from each identified protein is displayed. D) A metagene plot demonstrating CLIPseq profiles of eIF4E2^HA^-enriched and size-matched input samples. E) Analysis of eIF4E2^mAID-HA^ depletion over time upon addition of 500 µM IAA by western blot. The blot was probed with α-SAG1 as a loading control. Molecular weights are indicated in kDa. F) Plaque assay of vehicle (0.5% DMSO)-treated parasites, parasites pulsed for 24 h with 500 µM IAA or parasites treated with IAA for 6 days. Mean plaque numbers ± standard deviation were tested for statistical significance by a one-way ANOVA followed by Student’s t-test assuming unequal variances. ns = not significant. G) Analysis of parasite replication after a 16 h treatment with 500 µM IAA. Mean number of parasites per vacuole with standard deviation is shown. Statistical significance of the change between the mean number of parasites per vacuole was determined by Student’s t-test assuming unequal variances. ns = not significant.

**Figure S3. eIF4E1 binds mRNA 5’ caps with low selectivity.** A) eIF4E1 engagement with mRNAs is proportional to mRNA abundance as measured by CLIPseq and RNAseq. B) eIF4E1 engagement with mRNA is proportional to abundance of its translation as measured by CLIPseq and RIBOseq. C) The degree of translation largely correlates to mRNA abundance as measured by RIBOseq and RNAseq. Spearman correlation coefficient is presented on the bottom right of each graph. Discordance can be seen by apicoplast-encoded genes (purple) which are transcribed and translated within the organelle. Genes displaying reduced translational efficiency upon eIF4E1^mAID-HA^ depletion are marked in orange.

**Figure S4. Determination of 4EGI-1 activity against *Toxoplasma* and human fibroblasts.** Estimation of the 50% maximal effective concentration of 4EGI-1 against confluent human foreskin fibroblasts was assessed by alamar blue assay. Estimation of the 50% maximal effective concentration of 4EGI-1 against an RH strain *Toxoplasma* line that expresses a β-galactosidase reporter was determined by measuring the conversion of chlorophenol red-beta-D-galactopyranoside. Error bars represent the standard deviation between the replicates.

**Figure S5. Generation of eIF4E1^mAID-HA^ parasites in the ME49 background.** A) Analysis of eIF4E1^mAID-HA^ depletion by western blot in ME49 parasites upon addition of 500 µM IAA for 4 h. The blot was probed with α-SAG1 as a loading control. Molecular weights are indicated in kDa. B) Immunofluorescence microscopy of eIF4E^mAID-HA^ ME49 parasites upon treatment with DMSO (vehicle) or IAA for 4 h. DNA is stained with DAPI. Microscopy scale bar = 2 µm. C) Analysis of parasite replication after a 24 h treatment with 500 µM IAA. Mean number of parasites per vacuole with standard deviation is shown. Statistical significance of the change between the mean number of parasites per vacuole was determined by Student’s t-test assuming unequal variances. *p ≤ 0.001

**Figure S6. eIF4E1^mAID-HA^ expression is reduced upon alkaline stress.** A) Analysis of eIF4E1^mAID-HA^ expression by western blot in ME49 strain parasites after 5 days of alkaline stress without IAA treatment. The blot was probed with α-Aldolase as a loading control, as well as α-SAG1 and α-BAG1 as tachyzoite and bradyzoite specific markers, respectively. B) Densitometric measurements of eIF4E1^mAID-HA^ expression compared to aldolase from three biological replicates. *p ≤ 0.01

# Supplemental Table Legends

**Table S1.** eIF4E interacting proteins in tachyzoites as determined by immunoprecipitation followed by mass spectrometry analysis.

**Table S2.** Sites of eIF4E-mRNA interactions in tachyzoites as determined by CLIPseq.

**Table S3.** Differential gene expression at the transcriptional and translational level upon eIF4E1^mAID-HA^ depletion in tachyzoites as determined by RIBOseq with paired RNAseq.

**Table S4.** Differential gene expression after 24h and 48h depletion of eIF4E1^mAID-HA^ from RH and ME49 parasites.

**Table S5.** List of oligonucleotides and antibodies used in this study.
